# Rubisco Assembly in the Chloroplast

**DOI:** 10.3389/fmolb.2018.00024

**Published:** 2018-03-13

**Authors:** Anna Vitlin Gruber, Leila Feiz

**Affiliations:** ^1^Department of Molecular, Cell and Developmental Biology, University of California, Los Angeles, Los Angeles, CA, United States; ^2^Boyce Thompson Institute, Cornell University, Ithaca, NY, United States

**Keywords:** Rubisco, folding, assembly, chaperone, chaperonin, chloroplast

## Abstract

Ribulose-1,5-bisphosphate carboxylase/oxygenase (Rubisco) catalyzes the rate-limiting step in the Calvin-Benson cycle, which transforms atmospheric carbon into a biologically useful carbon source. The slow catalytic rate of Rubisco and low substrate specificity necessitate the production of high levels of this enzyme. In order to engineer a more efficient plant Rubisco, we need to better understand its folding and assembly process. Form I Rubisco, found in green algae and vascular plants, is a hexadecamer composed of 8 large subunits (RbcL), encoded by the chloroplast genome and 8 small, nuclear-encoded subunits (RbcS). Unlike its cyanobacterial homolog, which can be reconstituted *in vitro* or in *E. coli*, assisted by bacterial chaperonins (GroEL-GroES) and the RbcX chaperone, biogenesis of functional chloroplast Rubisco requires Cpn60-Cpn20, the chloroplast homologs of GroEL-GroES, and additional auxiliary factors, including Rubisco accumulation factor 1 (Raf1), Rubisco accumulation factor 2 (Raf2) and Bundle sheath defective 2 (Bsd2). The discovery and characterization of these factors paved the way for *Arabidopsis* Rubisco assembly in *E. coli*. In the present review, we discuss the uniqueness of hetero-oligomeric chaperonin complex for RbcL folding, as well as the sequential or concurrent actions of the post-chaperonin chaperones in holoenzyme assembly. The exact stages at which each assembly factor functions are yet to be determined. Expression of *Arabidopsis* Rubisco in *E. coli* provided some insight regarding the potential roles for Raf1 and RbcX in facilitating RbcL oligomerization, for Bsd2 in stabilizing the oligomeric core prior to holoenzyme assembly, and for Raf2 in interacting with both RbcL and RbcS. In the long term, functional characterization of each known factor along with the potential discovery and characterization of additional factors will set the stage for designing more efficient plants, with a greater biomass, for use in biofuels and sustenance.

## Introduction

Ribulose-1,5-bisphosphate carboxylase/oxygenase (Rubisco) is Earth's most abundant enzyme, used by autotrophic organisms to convert CO_2_ into organic compounds via the Calvin-Benson pathway (Andersson and Backlund, 2008). Rubisco catalyzes photosynthetic carbon reduction and photorespiratory carbon oxidation upon reaction with its substrates riboluse-1,5-bisphosphate, and CO_2_ or O_2_, respectively. The poor catalytic properties of Rubisco CO_2_ fixation necessitate a high abundance of this enzyme. Hence, Rubisco constitutes ~30–50% of the soluble protein in C_3_ plant leaves (Feller et al., 2008; Phillips and Milo, 2009). This enormous investment of energy, water and nitrogen limits biomass and crop yields.

Since all biomass results from the act of Rubisco in photosynthesis, increasing crop yields ultimately depends on improving the efficiency of carbon fixation. Although the catalytic performance of bacterial and archaeal Rubisco was successfully enhanced (Durão et al., 2015; Wilson et al., 2016), efforts to engineer a more catalytically efficient plant Rubisco remain unsuccessful (Parry et al., 2013). Consequently, not only has Rubisco become an intriguing model for studying protein folding and assembly, but also, elucidating the process of its biogenesis should allow researchers to improve its efficiency.

In order to engineer plant Rubisco or transplant a more productive version into hosts of agricultural or biotechnological interest, this protein should be viewed as a multi enzyme complex, in which all the parts work together and cannot be excluded (John Andrews and Whitney, 2003; Erb and Zarzycki, 2018). This review focuses on what is known about the folding and assembly of plant Rubisco. The chloroplast system supporting Rubisco biogenesis is unique in its complexity, and only the precise orchestration of folding and assembly leads to functional protein.

## Rubisco: an evolutionary perspective

Why is Rubisco so inefficient? Rubisco evolved before the oxygenation of the atmosphere, conditions under which there was no need to discriminate between O_2_ and CO_2_. In addition to the carboxylation, Rubisco catalyzes a non-productive oxygenation reaction that results in the formation of 2-phosphoglycolate (2PG). 2PG being a toxic compound, is recycled in plants in an energy-wasteful process called photorespiration (Zhu et al., 2010; Walker et al., 2016). The rise of atmospheric O_2_ concentration resulted in an increased error rate and forced Rubisco to lower its catalytic rate, reaching the Pareto optimality of enzyme activity and specificity (Tcherkez et al., 2006; Savir et al., 2010; Studer et al., 2014; Tawfik, 2014; Shih et al., 2016). The evolutionary adaptations eventually led to the formation of what is known as the “Rubiscosome”—a multifaceted complex of proteins which support Rubisco formation and function (Erb and Zarzycki, 2018). During this process, Rubisco evolved to form complex oligomeric structures and to collaborate with specific chaperones and activases.

**Figure 1:**
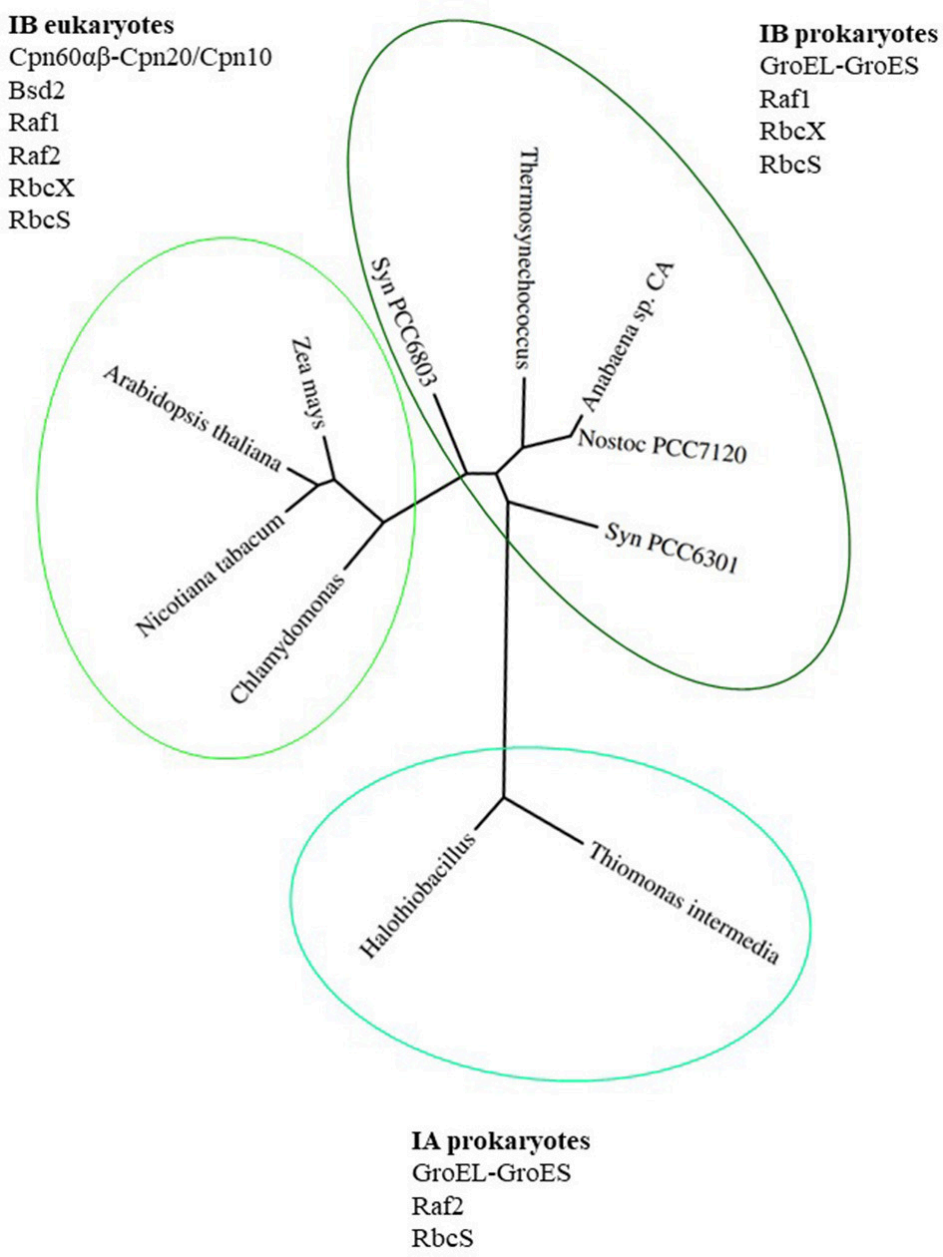
Phylogenetic tree of green-type RbcL subunits together with factors involved in Rubisco holoenzyme formation. Phylogenetic tree of RbcL sequences represents organisms mentioned in this review. The variety of folding and assembly factors and their involvement in Rubisco biogenesis are shown for each clade and discussed in the text. Species full names: *Thiomonas intermedia K 12, Halothiobacillus neapolitanus, Synechococcus PCC6301, Nostoc* sp. *PCC7120, Anabaena* sp. CA, *Thermosynechococcus elongatus, Synechocystis PCC 6803, Chlamydomonas reinhardtii, Arabidopsis thaliana, Zea mays, Nicotiana tabacum*. The phylogenetic tree was created using phylogeny.fr (http://www.phylogeny.fr; Dereeper et al., 2008, 2010).

Proteins belonging to the Rubisco family can be classified into 3 forms. The most ancient form III Rubisco, which is found in archaea, catalyzes regeneration of Ribulose-1,5-bisphosphate (RuBP), produced during nucleotide metabolism (Tabita et al., 2008a,b). In contrast, forms II and I evolved to catalyze RuBP carboxylation or oxygenation in an autotrophic, photosynthetic context. Form II Rubiscos are present in bacteria and dinoflagellates, while form I exists in plants, algae, cyanobacteria and proteobacteria (Andersson and Backlund, 2008). Form I Rubiscos are classified into red-type (in photosynthetic bacteria and non-green algae) and green-type (in proteobacteria, cyanobacteria, green algae and land plants) (Tabita, 1999; Badger and Bek, 2008; Tabita et al., 2008b). The green-type Rubiscos are further classified as forms IA and IB (Bracher et al., 2017). A phylogenetic tree of green-type Rubisco large subunits from various organisms mentioned in this review is presented in Figure 1, together with the factors participating in the assembly process.

The common feature of all Rubiscos is the formation of the active site at the interface between L_2_ - two Rubisco large subunits (RbcL, 50–55 kDa). Form II and III Rubiscos have (L_2_)_*n*_ stoichiometry (with *n* up to 5) while form I Rubisco is organized in four L_2_ dimers that assemble together with eight small subunits (RbcS, 12–18 kDa) to form a hetero-hexadecameric complex—L_8_S_8_. Rubiscos structure and function is extensively reviewed in Andersson and Backlund (2008) and Bracher et al. (2017). This higher-order oligomerization and presence of small subunits allowed for an increase in catalytic efficiency and substrate specificity. The increase in specificity for CO_2_ over O_2_ made Rubisco more vulnerable to inhibition by naturally occurring sugar phosphates, including RuBP (Mueller-Cajar, 2017). Evolutionary compensation took place in the form of Rubisco activases, which evolved to overcome this obstacle by releasing the inhibitory sugars (Salvucci et al., 1987; Mueller-Cajar et al., 2011; Tsai et al., 2015; Loganathan et al., 2016).

Form II Rubisco, which is composed only of two large subunits, can undergo spontaneous assembly in *E. coli* or *in vitro* without the assistance of GroEL and GroES (Goloubinoff et al., 1989a). Co-expression of the Rubisco subunits from *Rhodospirillum rubrum* along with GroEL-GroES in *E. coli*, however, significantly increased the assembly yield, suggesting that the folding machinery was a rate limiting factor (Goloubinoff et al., 1989b). In contrast, reconstitution of the cyanobacterial form I Rubisco from *Synechococcus PCC6301* (*Syn 6301*) with the assistance of GroEL-GroES chaperonins, yielded only small amount of holoenzyme until the assembly chaperone, RbcX was added, following RbcL folding (Liu et al., 2010).

Similar to their endosymbiont cyanobacterial ancestor, chloroplasts contain a form I Rubisco. Nevertheless, assembly of the chloroplast Rubisco has emerged as one of the most complicated assembly processes that is known for oligomeric proteins. Spontaneous assembly of the eight small and eight large subunits of form I Rubisco from any plant by random collision proved inefficient, both in *E. coli* and in a test tube, regardless of chaperonins and RbcX presence (Feiz et al., 2012; Hauser et al., 2015a, reviewed in Bracher et al., 2017). Bundle sheath defective 2 (Bsd2) was the first Rubisco specific factor that was shown to have an indispensable role in plant Rubisco assembly (Brutnell et al., 1999). Recently, forward genetics was used to identify two novel factors involved in plastid Rubisco biogenesis, Rubisco accumulation factor 1 (Raf1) (Feiz et al., 2012) and Rubisco accumulation factor 2 (Raf2) (Feiz et al., 2014). Structural and molecular characterization of these factors paved the road to elucidation of their role in Rubisco assembly, resulting in a successful expression of plant Rubisco holoenzyme in *E. coli* (Aigner et al., 2017). In the following chapters each factor will be described and its role in Rubisco biogenesis will be discussed.

## Chloroplast chaperonins

In eukaryotes, Rubisco large subunit is universally encoded by the chloroplast genome. The small subunits are encoded in the nucleus in plants and green algae and in the chloroplast genome in non-green algae (Tabita, 1999). Once transcribed and translated, the small subunit is imported into the chloroplast and folded to its functional form (Dobberstein et al., 1977; Highfield and Ellis, 1978). The large subunit is transcribed in the chloroplast, but to keep up a tight stoichiometry with its nucleus-encoded partner, its translation undergoes an assembly-dependent autoregulation (Wostrikoff and Stern, 2007).

One of the early post-translational chaperones in the process of Rubisco holoenzyme folding and assembly is the chloroplast chaperonin machinery. Chaperonins were initially discovered as a high-molecular-weight complex associated with RbcL, following its synthesis in isolated intact chloroplasts, prior to formation of holoenzyme (Barraclough and Ellis, 1980; Roy et al., 1982; Roy, 1989; Ellis, 1990). Early studies demonstrated that the protein was an oligomer composed of two subunit types, which reversibly dissociated into monomers in the presence of ATP, and was homologous to certain bacterial proteins that were crucial for phage morphogenesis (Hemmingsen et al., 1988). The general concept of a chaperone protein was born from these discoveries, and most research in the field focused on the extremely stable *E. coli* chaperonin system (GroEL-GroES).

Chloroplast homologs together with bacterial and mitochondrial chaperonins belong to the type I category. The type I chaperonin system consists of 2 oligomeric partners, working together to bind and fold partially denatured proteins. In *E. coli*, the binding partner is a tetradecamer of 60 kDa Cpn60 subunits (GroEL) while the co-chaperonin partner is a heptamer of 10 kDa Cpn10 subunits (GroES).

Though chloroplast chaperonins diverge from the bacterial system in several aspects, the most intriguing is the broad array of subunit types and the complexity of their oligomeric arrangements. Two GroEL-like subtypes are found in chloroplast, Cpn60α and Cpn60β, that can form homo- or hetero-oligomeric chaperonin species (Musgrove et al., 1987; Martel et al., 1990; Nishio et al., 1999). These subtypes are ~50% homologous to each other as well as to GroEL. Several paralogous forms of each type can be found in most plants (Hill and Hemmingsen, 2001; Schroda, 2004; Friso et al., 2010; Trösch et al., 2015). Similarly, chloroplasts harbor two types of co-chaperonin homologs. The first is a typical, GroES-like Cpn10, while the second gene is unique to chloroplast and consists of two Cpn10-like sequences joined head-to-tail with molecular weight of 20–23 kDa (Cpn20) (Bertsch et al., 1992). Similar to the 60 kDa partner, each chloroplast co-chaperonin also exists in several paralogous forms (Hill and Hemmingsen, 2001; Tsai et al., 2012). The entire cohort of Rubisco folding and assembly factors from *Arabidopsis thaliana* (*At—Arabidopsis*), *Zea mays* (*Zm—*maize), and *Chlamydomonas reinhardtii* (*Cr—Chlamydomonas*) are summarized in Table 1.

**Table 1:** Paralogs of Rubisco folding and assembly chaperones^*^.

**Subunit**	**MW (kDa)**	***Arabidopsis***	**Maize**	***Chlamydomonas***
RbcL	50–55	**AtCg00490**	GRMZM2G448344	CreCp.g007100
RbcS	12–18	**At5g38410** At1g67090 At5g38420 At5g38430	GRMZM2G113033 GRMZM2G098520	Cre02.g120150 Cre02.g120100
Cpn60α	~60	**At2g28000(**α**1)**	AC215201.3 GRMZM2G434173	Cre04.g231222
		At5g18820(α2)	GRMZM2G321767	Cre06.g309100
Cpn60β	~60	**At1g55490(**β**1)**	GRMZM2G083716	Cre07.g339150
		At3g13470(β2)		
		At5g56500(β3)	GRMZM2G015989	Cre17.g741450
		At1g26230(β4)	GRMZM2G042253	
Cpn10	~10	At2g44650(1) At3g60210(2)	GRMZM2G050961 GRMZM2G035063 GRMZM2G013652	Cre16.g673729
Cpn20	20–23	**At5g20720**	GRMZM2G091189 GRMZM2G127609 GRMZM2G399284	Cre08.g358562 Cre12.g505850
RbcX	~15	At4g04330(1)	GRMZM2G115476 NM_001149531	Cre07.g339000 Cre01.g030350
		**At5g19855(2)**		
Raf1	40–46	At5g28500(1) **At3g04550(2)**	GRMZM2G457621	Cre06.g308450
Raf2	~18	**At5g51110**	GRMZM2G139123	Cre01.g049000
Bsd2	~8	**At3g47650**	GRMZM2G062788	Cre06.g251716

* *Highlighted in bold are the subunits supporting Arabidopsis Rubisco expression and assembly in E. coli (Aigner et al., 2017)*.

Two oligomeric forms of Cpn60 were reconstituted *in vitro* from purified Cpn60α and Cpn60β monomers of several species (Dickson et al., 2000; Vitlin et al., 2011; Tsai et al., 2012; Bai et al., 2015) and were shown to form oligomers when expressed in *E. coli* (Cloney et al., 1992a,b; Bai et al., 2015). The reconstituted oligomers included the αβ hetero-oligomers, consisting of an approximate 1:1 ratio of α:β (Tsai et al., 2012) and all β homo-oligomers (Dickson et al., 2000; Vitlin et al., 2011; Bai et al., 2015). The αβ hetero-oligomers were further demonstrated to contain complicated mixtures of α and β paralogs (Peng et al., 2011; Bai et al., 2015; Ke et al., 2017).

By way of contrast, Cpn60α subunits expressed alone in *E. coli*, were not capable of assembling into a tetradecamer, nor were they able to form functional oligomers *in vitro* (Cloney et al., 1992a,b; Dickson et al., 2000; Bai et al., 2015). Domain swapping analysis in *Chlamydomonas* chaperonins demonstrated that equatorial domain controls the Cpn60α monomeric state. ATP hydrolysis drives allosteric rearrangement and promotes oligomer disassembly through Cpn60β C-terminal fragment, and cooperation from both subunits is needed to form active hetero-oligomers (Zhang et al., 2016a). Furthermore, functional divergence between the three *Chlamydomonas* subunits was attributed to both the apical and the equatorial domains, with both types of subunits evolved to have substrate specificity as well as co-chaperonin preference (Zhang et al., 2016b). Overall, Cpn60 complex formation from protomers *in vitro* depends critically on the presence of Mg-ATP, subunit concentration, temperature and Cpn60β protomer presence, suggesting that Cpn60β subunits likely initiate the oligomerization (Bloom et al., 1983; Lissin, 1995; Viitanen et al., 1998; Dickson et al., 2000; Bonshtien et al., 2009; Bai et al., 2015; Vitlin Gruber et al., 2018).

Significant heterogeneity was demonstrated for co-chaperonins as well. Cpn20 proteins from various organisms were shown to form tetrameric ring-like structures *in vitro* (Bertsch et al., 1992; Baneyx et al., 1995; Viitanen et al., 1995; Koumoto et al., 1999; Bonshtien et al., 2007; Tsai et al., 2012; Vitlin Gruber et al., 2013b, 2014; Bai et al., 2015). It was also demonstrated that *Arabidopsis* Cpn10(1) (At2g44650) organized into a ring of seven 10-kDa subunits, similar to GroES (Koumoto et al., 2001; Sharkia et al., 2003). In contrast, *Chlamydomonas* Cpn10 and Cpn23 proteins were purified as monomers (Tsai et al., 2012), and the third co-chaperonin from *Arabidopsis* Cpn10(2) (At3g60210), was purified as inactive, low molecular weight species (monomers or dimers) (Vitlin Gruber et al., 2014). Upon mixing Cpn10 with Cpn20 subunits, different active hetero-oligomeric species are produced *in vitro*. Interestingly, co-chaperonin subunits that are unable to support chaperonin function on their own, contributed to activity when incorporated into hetero-oligomer (Tsai et al., 2012; Vitlin Gruber et al., 2014; Guo et al., 2015). Even more interesting was the fact that co-chaperonins designed to contain either 6, 7, or 8 domains were fully functional with GroEL and Cpn60 oligomers, indicating that a symmetrical match is not stringently required for chaperonin function in general (Guo et al., 2015), though each co-chaperonin paralog might be crucial for folding of specific substrate. The latest progress in chloroplast chaperonin field is reviewed in Zhao and Liu (2018).

## Chaperonin subunit specificity and RbcL folding

The ability of Cpn60 and Cpn10 subunits to oligomerize in different combinations imply on a tremendous number of potential combinatorial Cpn60-Cpn10 pairs in the chloroplast, which could allow for a large number of substrates and modes of regulation (Vitlin Gruber et al., 2013a). Considering the heterogeneity, plasticity and asymmetry of the chloroplast chaperonin system, one can imagine chaperonin machines that are custom-made in a kind of substrate-directed organization. The importance of various subunits for folding of specific substrates is slowly being unraveled (reviewed in Vitlin Gruber et al., 2013a). Recent works in *Arabidopsis* demonstrated the specific role of Cpn60α2 (At5g18820) in folding of KASI (β-ketoacyl-[acyl carrier protein] synthase I) (Ke et al., 2017), and Cpn60β4 (At1g26230) was shown to be specifically required for the folding of NdhH, a subunit of the NADH dehydrogenase-like complex (NDH) (Peng et al., 2011).

But what do we know about chaperonin specificity for the most abundant chloroplast protein, RbcL? In maize, RbcL was found in association with a chaperonin complex composed of the two most abundant Cpn60 subunits, ZmCpn60α1 (Cps2 encoded by AC215201.3) and ZmCpn60β1 (GRMZM2G083716) (Feiz et al., 2012). Similarly, hetero-oligomer containing the most highly expressed Cpn60 subunits from *Arabidopsis* chloroplast (Cpn60α1—At2g28000 and Cpn60β1—At1g55490) efficiently folded the cognate AtRbcL subunit expressed in *E. coli*. Chaperonin activity could be facilitated by chloroplast tetrameric AtCpn20, as well as bacterial heptameric GroES, but not by chloroplast heptameric AtCpn10(1), suggesting a specificity of the later co-chaperonin in folding chloroplast substrates other than RbcL (Aigner et al., 2017). AtCpn60β1, which easily oligomerizes to form homo-tetradecamers (Cloney et al., 1992b; Vitlin et al., 2011), mediated RbcL folding in *E. coli* assisted by AtCpn20, albeit with lower efficiency in comparison to hetero-oligomer (Aigner et al., 2017). In the future it will be interesting to investigate the substrate specificity of additional chloroplast chaperonin paralogs and whether other Cpn60-Cpn10 pairs with various combination of subunits will be able to efficiently fold RbcL.

Numerous mutational analyses suggest that the Cpn60α subunit has a specific significance for the folding of RbcL. Examination of the data in the literature shows a correlation between down-regulation of specific chloroplast Cpn60α subunits and the amount of Rubisco (Vitlin Gruber et al., 2013a). It should be noted that unfolded or unassembled Rubisco cannot accumulate in plants and is completely prone to degradation, so Rubisco content in alpha mutants is not only the indicator of Rubisco synthesis, but of its folding and assembly as well. For example, the maize *cps2* mutant exhibited a pale green and seedling-lethal phenotype with 95% less Rubisco than wild type, while the level of other chloroplast proteins remained intact (Feiz et al., 2012), suggesting Rubisco specificity of this ZmCpn60α1. Mutation in the *cps2* ortholog of rice (Os12g17910), also resulted in drastically reduced levels of RbcL in a pale green seedling, without a decrease in the levels of other important proteins (Kim et al., 2013). A single amino acid substitution (D335A) at a conserved position in *Arabidopsis* ortholog Cpn60α1, caused retarded growth and pale green-leaf phenotype. Although the total levels of Cpn60α and Cpn60β were increased in this mutant, possibly due to compensation effects, the levels of RbcL were reduced (Peng et al., 2011). Recently, two new *Arabidopsis* and rice mutants carrying mutations in Cpn60αs were described. In *Arabidopsis*, mutation in Cpn60α1 (At5g18820) caused embryo development arrest at the globular stage (Ke et al., 2017). Rice thermo-sensitive chloroplast development 9 (*tcd9*) mutant grown below 24°C, had an albino phenotype at the 3-leaf stage (Jiang et al., 2014). It remains to be determined whether these Cpn60α subunits are involved in Rubisco folding.

What is the precise role of the Cpn60α subunit in RbcL folding? Structural studies in *Chlamydomonas* indicated that the Cpn60α apical domain recognizes CrRbcL with higher efficiency in comparison to Cpn60β, but it comes with the price of hindered functional co-operation of Cpn60α with different co-chaperonins (Zhang et al., 2016b). Based on these results we could hypothesize that Cpn60α evolved to specifically recognize and perhaps prioritize RbcL binding in the chloroplast, while Cpn60β maintained the responsibility for oligomerization and productive interaction with co-chaperonins. Characterization of additional chaperonin mutants will reveal the list of chaperonin subunits specifically required for Rubisco folding, as well as their specificity for other chloroplast substrates, while additional biochemical studies will help uncovering the precise mode of function of chloroplast chaperonins.

## RbcX enhances RbcL_8_ assembly by stabilizing folded RbcL_2_

RbcX gene was first described in cyanobacterium *Anabaena 7120* (*Nostoc* sp. *PCC7120*) (Larimer and Soper, 1993) and its role was gradually revealed in subsequent studies. RbcX is conserved from the cyanobacteria to plants (Hauser et al., 2015b). Co-expression of the RbcX genes from various cyanobacteria as well as from *C. reinhartii* or *A. thaliana*, was shown to enhance the assembly of cyanobacterial Rubisco in *E. coli* (Li and Tabita, 1997; Onizuka et al., 2004; Saschenbrecker et al., 2007; Kolesinski et al., 2011; Bracher et al., 2015), suggesting a conserved mode of function for all the homologs. Insertional inactivation of RbcX genes that were located in or outside of the Rubisco operons in two cyanobacteria strains, suggested that the RbcX protein may be essential for Rubisco biogenesis only when it is expressed from the Rubisco operon (Li and Tabita, 1997; Emlyn-Jones et al., 2006). Considering the large diversity of RbcL genes from different cyanobacterial strains, as presented in Figure 1, it seems that some developed dependence on RbcX assistance, while others are RbcX independent, or in need of other assembly factors.

RbcX is a homodimer of a ~15 kDa subunits, mostly α-helical. In *Syn 6301*, each RbcX subunit binds to a motif at the C-terminus of a folded large subunit, thereby clamping together the RbcL antiparallel dimer. The term assembly chaperone was coined for RbcX because of the mechanism by which this protein mediates the oligomeric assembly. By stabilizing the RbcL dimeric core, RbcX_2_ prevents rebinding of the labile, partially folded RbcL monomers to GroEL-GroES, and facilitate their assembly into the RbcL_8_ core complex. Finally, RbcS binding to RbcL_8_ triggers a conformational change that results in RbcX release and formation of the holoenzyme (Saschenbrecker et al., 2007; Liu et al., 2010). The ease by which RbcS replaces RbcX during assembly originates from the dynamic nature of the RbcX interaction with RbcL. When high affinity, heterologous RbcX (from *Anabaena* sp. CA) was co-expressed with RbcL in *E. coli*, the RbcX could not be replaced by RbcS. This phenomenon originally facilitated determination of the RbcX-RbcL structure (Saschenbrecker et al., 2007), and led to successful reconstitution of the holoenzyme from *Syn 6301* (Liu et al., 2010).

*Arabidopsis* contains two RbcX genes. AtRbcX2, encoded by the At5g19855 gene is closely related to the cyanobacterial homolog, and was found in the stromal fraction, while AtRbcX1, encoded by the At4g04330 gene, is a more distant homolog and was shown to localize in the thylakoid fraction (Kolesinski et al., 2011). Both proteins were crystallized and shown to have different affinities for the RbcL C-terminus (Kolesinski et al., 2013). AtRbcX2 was one of the assembly factors that when expressed with chaperonins and other assembly chaperones in *E. coli*, resulted in the *Arabidopsis* Rubisco formation. This protein, however, was suggested to be more of an enhancer than an essential chaperone, since in its absence, around 50% of recombinant Rubisco was formed (Aigner et al., 2017). The evolutionary perspective of the RbcX gene duplication in plants and the relevance of this duplication to Rubisco biogenesis is another intriguing question. The thylakoid localization of AtRbcX1 together with its lower affinity toward RbcL (Kolesinski et al., 2013), may suggest a divergent role for this homolog. Interestingly, *Chlamydomonas* encodes only the AtRbcX1 homologs, CrRbcXA and CrRbcXB. CrRbcXA was structurally and functionally characterized and shown to support cyanobacterial Rubisco assembly (Bracher et al., 2015). In the future, characterization of RbcX mutants as well as additional biochemical studies could reveal their precise role in Rubisco assembly and the unique properties of each homolog.

## Raf1 is essential for RbcL assembly, downstream of chaperonin folding

Rubisco accumulation factor 1 (Raf1), the first factor characterized as an assembly chaperone involved in Rubisco biogenesis in chloroplasts (Feiz et al., 2012), was found by screening the maize Photosynthetic Mutant Library (PML), a collection of ~2,000 photosynthetic mutants, for Rubisco-specific deficiencies (Belcher et al., 2015). The maize *raf1* mutants are pale green, unable to accumulate Rubisco and are lethal at the seedling stage. Characterization of the mutant indicated that in the absence of Raf1, newly-synthesized RbcL subunits are not assembled into the holoenzyme, but instead are trapped in an ~800 kDa chaperonin complex (Feiz et al., 2012). Even though co-immunoprecipitation of RbcL with Raf1 indicated that Rubisco is the primary protein client of the Raf1, these experiments could not reveal a detailed mode of action of Raf1 in the chloroplast.

Functional characterization of cyanobacterial Raf1 from *Thermosynechococcus elongatus* (*Te*) indicated that it forms intermediate complexes with RbcL, resembling the RbcX role (Kolesinski et al., 2014; Hauser et al., 2015a). *In vitro* reconstitution showed that two RbcL-Raf1 complexes, Raf1_2_-RbcL_2_ and Raf1_8_-RbcL_8_, were formed in the presence of the GroEL and GroES. Similar to RbcX, Raf1 in the octameric complex was displaced by RbcS to complete the assembly of the holoenzyme (Hauser et al., 2015a). Mutational analysis of the C- and N- terminal domains of the cyanobacterial Raf1 showed that Raf1 binds to RbcL at different interaction sites than RbcX. It was also shown that unlike RbcX, the Raf1 α-domain and RbcS share overlapping binding sites on RbcL, causing the highly dynamic Raf1-RbcL interaction to allow RbcS binding (Hauser et al., 2015a). This could be the reason behind the difficulty of capturing the Raf1-RbcL intermediates in chloroplast lysate. Taking into consideration that RbcX was reported as being fully capable of assembling the cyanobacterial Rubisco (Saschenbrecker et al., 2007; Liu et al., 2010), the most plausible hypothesis for the Raf1 function in cyanobacteria is that it is redundant with RbcX in the assembly pathway. Indeed, a recent finding showed that similar to RbcX deletion in some cyanobacteria, Raf1 deletion in *Synechocystis PCC 6803* (*Syn 6803*) did not cause any growth defect (Kolesinski et al., 2017), suggesting that these factors might have overlapping functions.

Crystal structures of the N- and C-terminal domains of the *Arabidopsis* Raf1 suggested that plant Raf1 has a different structure than plant RbcX and consists of an N-terminal α-helical domain, and a C-terminal β-sheet domain connected by a flexible linker segment (Hauser et al., 2015a). In addition, plant Raf1 is essential for Rubisco assembly, while RbcX was shown to only enhance the assembly process (Aigner et al., 2017), suggesting that these chaperones might act sequentially, in parallel or in cooperation, rather than being redundant as in cyanobacteria.

A direct application of Raf1 discovery in crop improvement was implemented by taking advantage of Raf1 co-evolution with RbcL (Whitney et al., 2015). In this study transplastomic expression of AtRaf1 in the *Nicotiana tabacum* (*Nt*) host, which was deficient in native NtRbcL, but expressing a heterologous Rubisco, composed of the AtRbcL and NtRbcS, resulted in quicker production and increased levels of Rubisco, bigger plants and improved photosynthesis, relative to the same host expressing only the endogenous NtRaf1. The two-fold increase in Rubisco content in the presence of AtRaf1 was still half the level of holoenzyme in WT tobacco plants. Even though this was attributed to a five-fold lower AtRbcL transcript levels relative to the endogenous NtRbcL in the WT, it is likely that co-expression of the other cognate factors that have co-evolved with RbcL, including Raf2, Bsd2, RbcX, and chaperonin homologous, was essential for a full assembly of the heterologous Rubisco. The importance of Raf1 and RbcL co-evolution was demonstrated again, when *Arabidopsis* assembly factors were not compatible for folding recombinant NtRubisco, until Raf1 replacement with the cognate protein slightly improved the holoenzyme assembly (Aigner et al., 2017), suggesting the co-evolution of not only Raf1 but other members of the Rubiscosome, unique to each plant.

## Raf2 is essential for Rubisco biogenesis

The other Rubisco deficient mutant that was found in the maize PML was *raf* 2 (*rubisco accumulation factor* 2), which carries a loss of function mutation in the GRMZM2G139123 locus encoding a chloroplast-targeted protein with an inactive pterin-4a-carbinolamine dehydratase (PCD) domain (Feiz et al., 2014). Raf2 homologs are found in vascular plants, green algae and in bacteria that accumulate form IA Rubisco in their CO_2_-concentrating organelles called α-carboxysomes. Raf2 has not been found in the cyanobacterial strains that contain the plant-like form IB Rubisco, nor in red algae (Hauser et al., 2015b). Loss of Raf2 function results in a weaker phenotype than disruption of Raf1 in maize, nevertheless *raf2* is also seedling-lethal (Feiz et al., 2014). In the absence of Raf2, newly synthetized RbcL is associated with the chaperonin complex, suggesting that like Raf1, Raf2 functions at a post-chaperonin assembly stage (Feiz et al., 2014; Aigner et al., 2017).

Chemical cross-linking followed by co-immunoprecipitation showed that maize Raf2 interacts with RbcS and to a lesser extent with RbcL in the chloroplast stroma (Feiz et al., 2014). Recombinant maize Raf2 (~18 kDa) migrates as dimers and tetramers on native gels (Feiz et al., 2014), consistent with animal PCD proteins (Hevel et al., 2008), and with the Raf2 homolog from *Thiomonas intermedia* K12, which was crystallized as a dimer (Wheatley et al., 2014). In α-carboxysome-containing bacteria, such as chemoautotrophic bacterium *Thiomonas intermedia* K 12 and *Halothiobacillus neapolitanus*, Raf2 is expressed from the Rubisco operon and does not show PCD activity. Heterologous co-expression of Raf2 from the latter strain with Rubisco, GroEL and GroES in *E. coli*, increased the amount of assembled Rubisco (Wheatley et al., 2014). AtRaf2 was one of the assembly chaperones whose presence proved essential in assembling AtRubisco in *E. coli* (Aigner et al., 2017).

The mechanism by which Raf2 plays role(s) in Rubisco biogenesis has yet to be studied in detail. It has been known that animal PCD dimers mediate dimerization of the HNFα homeodomain transcription factor, a key step in HNFα activation (Endrizzi et al., 1995; Rose et al., 2004). Structural modeling of plant Raf2 indicated the conservation of an α-helical stretch of 17 amino acids that was proposed to function in both dimerization of the PCD and its interaction with HNFα, perhaps suggesting a dimerization or oligomerization role for Raf2 in Rubisco holoenzyme assembly (Feiz et al., 2014).

## Bsd2 is essential for Rubisco assembly by stabilizing RbcL_8_ intermediate

Bsd2 was identified as a plastid-localized DnaJ-like Zn finger-containing protein with a role in post-translational biogenesis of maize Rubisco. Like *raf1* and *raf2*, the *bsd2* mutant is Rubisco-deficient and seedling lethal. Originally, Bsd2 was proposed to be part of a complex containing DnaJ-like (Hsp40) and Dna-K like (Hsp70) proteins, hypothetically transferring the newly-synthesized RbcL to the chaperonin folding apparatus (Brutnell et al., 1999). However, there is no evidence to support this model or to suggest that chaperonin-assisted folding of RbcL is preceded by a Dna-J/Hsp70-mediated complex that can bind the emerging RbcL nascent chain and protect it from aggregation. Overall, Bsd2 similarity to Hsp40 is limited to the hairpin structure of the Zn finger domain general architecture (Aigner et al., 2017).

Bsd2 homologs are limited to the plant and algae lineages (Hauser et al., 2015b), suggesting their emergence after the endosymbiotic event and chloroplast evolution. Pulse-labeling of chloroplast proteins in the maize *bsd2* mutant showed that the newly synthesized RbcL is associated with the chaperonin complex, suggesting that like Raf1 and Raf2, Bsd2 functions at a post-chaperonin stage of Rubisco assembly (Feiz et al., 2014). Co-immunoprecipitation with maize Bsd2 occurred for RbcS and to a lesser extent with RbcL and occurred reciprocally with Raf1 (Feiz et al., 2014).

In some of the experiments that were conducted during in *E. coli* biogenesis of the chloroplast Rubisco (Aigner et al., 2017), two higher order complexes migrated above the Rubisco holoenzyme on native gel. Whereas none of these bands showed any trace of Raf1, Raf2, or RbcX, the higher band contained RbcL and Bsd2 and the lower contained RbcL, Bsd2, and RbcS. The disappearance of both bands along with the promotion in RbcL_8_S_8_ formation, after an increase in RbcS expression, suggested that the higher order Bsd2-bound complexes might have formed due to RbcS insufficiency. Interestingly, when RbcS was deleted from the co-expression experiment, only the higher band was observed and when both RbcS and Raf2 were omitted, none of complexes were detected (Aigner et al., 2017), suggesting that Raf2 mediates the Bsd2-RbcL interaction.

AtBsd2 alone crystallized as monomer of ~8 kDa (Aigner et al., 2017). In the center of its hairpin structure two Zn atoms were found, each coordinated by four cysteines. Because plant RbcL_2_ or RbcL_8_ intermediates have not been detected in *E coli*, cyanobacterial TeRbcL (from *Thermosynechococcus elongatus* BP-1) was co-expressed with AtBsd2 and the crystal structure of the TeRbcL_8_AtBsd2_8_ complex was obtained. In the complex, Bsd2 join RbcL dimers to form an RbcL_8_ core surrounded by eight Bsd2 proteins. The relevance of the AtBsd2-TeRbcL interacting residues was further validated by mutational analysis of AtBsd2 and testing its competency in assembling AtRubisco in *E. coli*. No overlap was observed for Bsd2 and RbcS binding sites on RbcL (Aigner et al., 2017). Cyanobacterial RbcX and Raf1 were also shown to bind to either TeRbcL_8_ or SeRbcL_8_ (from *Synechococcus elongatus*) (Bracher et al., 2011; Hauser et al., 2015a). The TeRbcL_8_AtBsd2_8_ complex, however, was suggested to be the last assembly intermediate before holoenzyme formation with RbcS (Aigner et al., 2017).

## Detailing the assembly pathway by *in vitro* reconstitution of plant Rubisco

**Figure 2:**
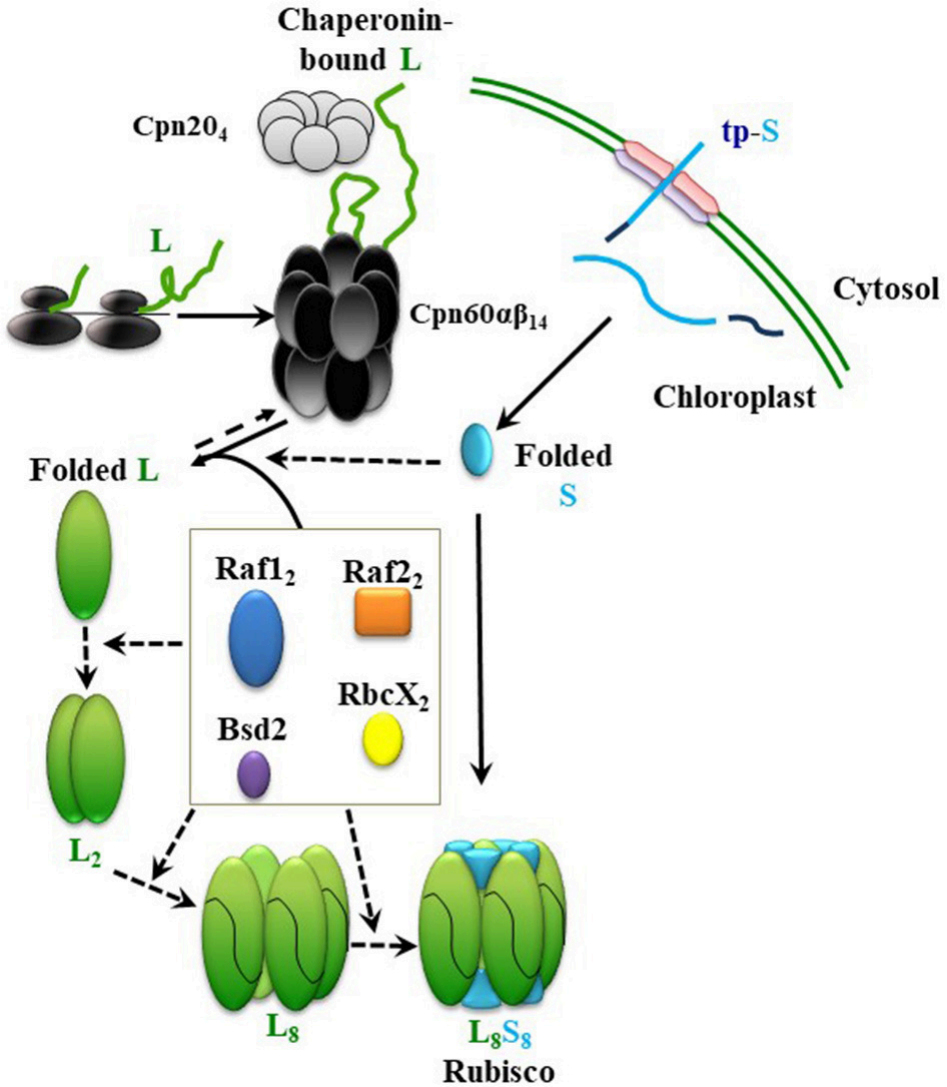
Model summarizing the roles of different chaperones in Rubisco assembly. From top; Newly-synthesized RbcL (L) interacts with the chaperonin complex, which leads to correct folding (Native L). After import into chloroplast and cleavage of its transit peptide, RbcS (S) folds spontaneously, or with the help of a chaperone. Raf1, Raf2, RbcX, and Bsd2 form dynamic intermediates with the folded RbcL. RbcS subunits could either displace the chaperones in a final chaperone-RbcL intermediate to form the holoenzyme (L_8_S_8_), or interact with chaperones and RbcL in earlier stages of the assembly. Continuous and dashed arrows indicate certain and speculative nature of each step, respectively.

Elucidation of the assembly steps of cyanobacterial Rubisco and identification of the essential chloroplast factors helped with partial depiction of the assembly pathway for plant Rubisco and led to successful expression of *Arabidopsis* Rubisco in *E. coli* (Aigner et al., 2017). A proposed path, leading to holoenzyme formation in chloroplasts, is described in Figure 2. In short, newly-synthesized RbcS (S) is imported into the chloroplast and folded, independently or with the help of chaperones, to the native state, after cleavage of its transit peptide. Newly-synthesized RbcL (L) in chloroplast is folded by the chaperonin hetero-complex assisted by Cpn20. In the absence of assembly factors, RbcL would not be able to escape from the chaperonin cycle, ultimately leading to aggregation and proteolysis. Raf1, Raf2, and RbcX dimers and Bsd2 monomers mediate formation of intermediates from folded RbcL, leading to their displacement by the RbcS and formation of the holoenzyme. So far, we have no evidence for the presence of any distinct post-chaperonin RbcL-containing intermediates, such as RbcL_2_ and RbcL_8_, that can be formed prior to biogenesis of the chloroplast holoenzyme. Putative intermediate complexes containing RbcS, RbcL, Raf1, Raf2, and Bsd2 were co-immunoprecipitated from plant lysates, following *in vivo* crosslinking, but their size, composition and stoichiometry remained to be determined (Feiz et al., 2014).

Using cyanobacterial RbcL, similar roles in dimerization and octamerization of the chloroplast RbcL have been proposed for RbcX, Raf1, and Bsd2 (Bracher et al., 2011; Hauser et al., 2015a; Aigner et al., 2017). In the most recent model, however, sequential functions have been proposed, during which Raf1 and RbcX are involved in the earlier RbcL oligomerization steps, and their replacement by Bsd2 mediates a later stabilization step of the RbcL_8_ core. According to this model, RbcS may only have to replace Bsd2 before formation of the holoenzyme (Aigner et al., 2017).

Many question marks surround this model. What is the precise role of Raf2? Is RbcS folded spontaneously or in need of chaperone assistance to reach conformation compatible for RbcL binding? Do RbcX and Raf1 act in parallel or cooperatively? How Bsd2 displaces Raf1/RbcX? How RbcS displaces Bsd2? Are there additional factors involved in Rubisco biogenesis? Revealing the sequential steps of assembly, as well as the precise role of different chaperone paralogs is the next challenge. Further *in vitro* and *in vivo* experiments seem essential in unraveling the assembly steps and characterizing the unique structural and functional properties of the different factors.

Reconstitution of *Arabidopsis* Rubisco *in vitro* was previously attempted. The results showed that RbcL subunits stayed bound to chaperonins and did not assemble into any type of oligomers or holoenzyme despite the presence of all assembly factors except Bsd2 (Hauser, 2016), as one would expect in light of the recent work. Whether the entire cohort of assembly factors, their exact levels, and an accurate timing of theirs functions, would be sufficient for *in vitro* assembly, is yet to be determined. Evolution has invested tremendous resources in the fine-tuning of various folding and assembly factors and their compatibility with RbcL and RbcS in chloroplast. Further genetic and biochemical studies are necessary for complete, in detail understanding of this complex pathway.

## Author contributions

All authors listed have made a substantial, direct and intellectual contribution to the work, and approved it for publication.

### Conflict of interest statement

The authors declare that the research was conducted in the absence of any commercial or financial relationships that could be construed as a potential conflict of interest.
